# Efficient algorithms for biological stems search

**DOI:** 10.1186/1471-2105-14-161

**Published:** 2013-05-16

**Authors:** Tian Mi, Sanguthevar Rajasekaran

**Affiliations:** 1 Department of Computer Science and Engineering, University of Connecticut, Storrs, CT, USA

## Abstract

**Background:**

Motifs are significant patterns in DNA, RNA, and protein sequences, which play an important role in biological processes and functions, like identification of open reading frames, RNA transcription, protein binding, etc. Several versions of the motif search problem have been studied in the literature. One such version is called the Planted Motif Search (PMS)or (*l, d*)-motif Search. PMS is known to be NP complete. The time complexities of most of the planted motif search algorithms depend exponentially on the alphabet size. Recently a new version of the motif search problem has been introduced by Kuksa and Pavlovic. We call this version as the *Motif Stems Search (MSS)* problem. A motif stem is an *l*-mer (for some relevant value of *l*)with some wildcard characters and hence corresponds to a set of *l*-mers (without wildcards), some of which are (*l, d*)-motifs. Kuksa and Pavlovic have presented an efficient algorithm to find motif stems for inputs from large alphabets. Ideally, the number of stems output should be as small as possible since the stems form a superset of the motifs.

**Results:**

In this paper we propose an efficient algorithm for MSS and evaluate it on both synthetic and real data. This evaluation reveals that our algorithm is much faster than Kuksa and Pavlovic’s algorithm.

**Conclusions:**

Our MSS algorithm outperforms the algorithm of Kuksa and Pavlovic in terms of the run time as well as the number of stems output. Specifically, the stems output by our algorithm form a proper (and much smaller)subset of the stems output by Kuksa and Pavlovic’s algorithm.

## Background

Motifs, or sequence motifs, are patterns of nucleotides or amino acids. Motifs are often related to primer selection, transcription factor binding sites, mRNA processing, transcription termination, etc. Sequence motifs of proteins are typically involved in functions such as binding to a target protein, protein trafficking, post-translational modifications, and so on. Motif search problem has been studied extensively due to its pivotal biological significance. Several types of algorithms have been proposed for motif search. In one such class of methods, putative motifs in an input biological query sequence are predicted based on a database of known motifs. Examples include [1-3]. In another class of methods, motifs are assumed to appear frequently in a set of sequences, like the same protein sequence from different species. Here the problem of motif search is reduced to that of finding subsequences that occur in many of the input sequences. Planted motif search (PMS)is one such formulation.

Numerous algorithms have been proposed to solve the PMS problem. The WINNOWER algorithm uses a graph to transform this problem into one of finding large cliques in the graph [4]. The PatternBranching algorithm introduces a scoring technique for all the motif candidates [5]. The PROJECTION algorithm repeatedly picks several random positions and uses a hash table with a threshold to limit the motif candidates [6]. Bailey 1995 [7] employs expectation maximization algorithms while Gibbs sampling is used in [8,9]. MULTIPROFILER [10], MEME [11], and CONSENSUS [12] are also known PMS algorithms.

An *exact* PMS algorithm always outputs all the motifs present in a given set of sequences. MITRA employs a mismatch tree structure to generate the motif candidates efficiently [13]. RISOTTO constructs a suffix tree to compare sequences [14]. PMS1 [15] considers all the motif candidates and evaluates them using sorting. Voting uses a hash table to locate the motifs [16]. PMS2 is based on PMS1 and it extends smaller motifs to get longer motifs, and PMS3 forms a motif of a desired length using two smaller motifs [15]. PMSPrune introduces a tree structure for the motif candidates and uses a branch-and-bound algorithm to reduce the search space [17]. PMS4 is a speed-up technique that finds a superset of the motifs using a subset of the input sequences and validates those candidates [18]. PMS5 employs an Integer Linear Programming (ILP)as the branch-bound algorithm for a speedup [19]. PMS6 uses the solutions of such ILPs to generate motif candidates [20].

Most of the work on exact algorithms for PMS has focussed on DNA or RNA sequences where |*Σ*|=4. Little work has been done on larger alphabet sizes, such as on proteins. A recent work focusses on protein sequences [21]. However, the stemming algorithm proposed in this paper does not solve the PMS problem. In particular, it does not find motifs but rather *motif stems*. A motif stem (denoted as stem from hereon)can be thought of as an *l*-mer (for some relevant value of *l*)with some wild cards present in it. As a result, a stem stands for a set of motifs. A stemming algorithm generates stems (or motifs with wildcards)to represent motifs for large-alphabet inputs [21]. The stemming algorithm of [21] generates a very large set of stems (and hence a very large superset of motifs). In this paper we propose two algorithms for Motif Stems Search, MSS1 and MSS2, which outperform the stemming algorithm of [21]. The new algorithms generate a much smaller set of stems. The stems generated by the algorithm of [21] as well as MSS1 and MSS2 are guaranteed to be supersets of all the motifs present in a given set of input sequences.

## Methods

### Motif search on large alphabets

In this section we provide some definitions pertinent to PMS and MSS problems.

#### Definition 1

A sequence *x*=*x*[1,2,…,*l*] (|*x*|=*l*)on *Σ* (*x*[*i*]∈*Σ*, 1≤*i*≤*l*)is an *l*-mer.

#### Definition 2

Given two *l*-mers *x* and *y*, the Hamming distance between two *l*-mer is defined as:

$$\text{HD}(x,y)=\left|\left\{i|x[i]\neq y[i],1\leq i\leq l\right\}\right|$$

#### Definition 3

Given an *l* mer *x* and a sequence *s* of length *m*, the Hamming distance between *x* and *s* is defined as

$$\text{HD}(x,s)=\underset{1\leq i\leq m-l+1}{\text{min}}\{\text{HD}(x,s[i,i+1,\ldots ,i+l-1])\}.$$

#### Definition 4

**(Planted Motif Search (PMS)Problem).** Let *S* be a set of *n* sequences of length *m* each on an alphabet *Σ*. Specifically, let *S*={*s*_*i*_||*s*_*i*_|=*m*, 1≤*i*≤*n*}. The *planted motif search problem* or *(l,d)motif search problem* takes as input *S* and two integers *l* and *d*, and finds every string *x* of length *l* such that for every *s*_*i*_, the Hamming distance between *x* and *s*_*i*_ is no more than *d*. In particular, we want to compute the following set:

$$\{x|\left|x\right|=l,\text{HD}(x,s_{i})\leq d,\;\text{for}\;1\leq i\leq n\}.$$

Any such *x* is called an (*l,d*)-motif. Any *l*-mer of *s*_*i*_ that is at a Hamming distance of ≤*d* from *x* is called *an occurrence or instance of x*.

#### Definition 5

Given an *l*-mer *x*, the *d*-neighbors of *x* is defined to be {*y* : |*y*|=*l* and *HD*(*x*,*y*)≤*d*}. The *d*-neighbors of *x* in any sequence *s* is defined to be the intersection of the *d*-neighbors of *x* and the set of *l*-mers in *s*.

#### Observation 1

If the Hamming distance between two *l*-mers *x*_1_ and *x*_2_ is larger than 2*d* (i.e., *HD*(*x*_1_,*x*_2_)≥2*d*)then no *l*-mer *x*_3_ exists such that *HD*(*x*_1_,*x*_3_)≤*d* and *HD*(*x*_2_,*x*_3_)≤*d*.

PMS algorithms are typically tested on random data with *n*=20 and *m*=600. Each input string is randomly generated such that each symbol in each string is equally likely to be any character from the alphabet. A motif is generated randomly. Randomly mutated versions of this motif are planted in the input strings (one mutated motif per string). For a given value of *l*, we call the pair (*l,d*)a *challenging instance* if *d* is the smallest value for which the number of (*l,d*)-motifs occurring in the input strings by random chance is ≥1. Some of the challenging instances are: (9, 2), (11, 3), (13, 4), (15, 5), (17, 6), (19, 7), and so on. One of the performance measures of interest for any exact algorithm is the largest challenging instance that it can solve. MITRA can solve the instance of (13, 4)[13], and RISOTTO [14] and Voting [16] successfully run on (15, 5). PMSPrune solves up to (19, 7)[17]. PMS5 [19] and PMS6 [20] can handle (23, 9). These statistics are based on DNA sequences where |*Σ*|=4.

The time complexities of exact algorithms typically depend exponentially on the size of *Σ*. PMS0 takes O(m2nlld|Σ|d) time, and PMS1 costs O(mnld|Σ|dlw) time where *w* is the word length of the computer [15]. It needs O(mn2ldld|Σ|d) time for RISOTTO [14], O(mnld|Σ|d) for Voting [16], and O(m2nld|Σ|d) for PMSPrune [17].

When the size of the alphabet is large (e.g., |*Σ*|=20 for proteins), the above exact algorithms will take a very long time. Kuksa and Pavlovic have introduced a new version of the motif search problem and proposed an efficient algorithm to solve it on large alphabets. A motif *stem* is an *l*-mer with wildcards. Thus a stem represents a set of *l*-mers without wildcards. For example, if *g* ∗ *acc* is a DNA stem, it represents the following 5-mers without wildcards: *ggaac*,*gcaac*,*gtaac*, and *gaaac*. Given a set of strings from some alphabet, the problem of finding motif stems in them is known as the *Motif Stem Search (MSS)* problem. We focus on MSS in this paper.

#### Definition 6

Motif Stem Search (MSS)Problem. Input are *N* sequences and two integers *l* and *d*. The problem is to find a set of stems such that the set of *l*-mers represented by these stems is a superset of all the (*l,d*)-motifs present in the *N* sequences.

As stated above, there are many possible solutions to the MSS problem. The challenge then is to come up with a superset as small as possible which covers all the (*l,d*)-motifs. In other words, we want the number of *l*-mers (without wildcards)represented by the stems to be as small as possible.

#### MSS1 - A basic Algorithm

Based on OBSERVATION 1, if the Hamming distance between an *l*-mer *x* and a sequence *s* is larger than 2*d*, then no *l*-mer *x*’ exists such that *HD*(*x*,*x*^′^)≤*d* and *HD*(*x*^′^,*s*)≤*d*. This leads us to the following observation.

##### Observation 2

Given an *l*-mer *x*, if ∃*s*_*i*_ such that *HD*(*x*,*s*_*i*_)>2*d*, then none of *x*’s *d*-neighbors can be a motif.

The stemming algorithm of [21] works as follows. It makes use of OBSERVATION 2 crucially. OBSERVATION 2 states that an *l*-mer *x* in any input string cannot be an instance of an (*l,d*)-motif if there exists at least one input string *s* such that *HD*(*x*,*s*)>2*d*. The algorithm of [21] first identifies a set *I* of possible motif instances. An *l*-mer *x* in any input string *s* will be included in *I* if and only if *HD*(*x*,*s*^′^)≤2*d* for every input string *s*’. Having found such a set *I*, the algorithm then uses *I* to generate stems. The stems are found as follows: For every *x*,*y*∈*I*, the algorithm generates the common *d*-neighbors of *x* and *y* as stems. The union of all such stems will constitute candidate motif stems. This union is a superset of the motif stems. Finally, for each candidate stem, the algorithm checks if this is a correct answer or not. All valid stems that pass this test are output.

In Algorithm 1 and Algorithm 2 we present a faster algorithm (than that of [21])for generating motif stems. In Algorithm 1 we present an algorithm for generating the set *I*. The cardinality of *I* that we generate is a much smaller subset of the *I* generated in the stemming algorithm of [21]. For any pair of *l*-mers (*x*,*x*^′^)in the set *I*, we begin with *x* and replace some characters in *x* with wildcards to generate MSS candidates. The positions in which *x* and *x*’ match are referred to as the *matching region* and the positions in which *x* and *x*’ differ are referred to as the *non-matching region*. The wildcards can be placed in the matching region and/or the non-matching region. Any stem *t* is generated by placing wildcards in *x*. Therefore, wildcards in the generated stem *t* are always treated as mismatches between *t* and *x*, independent of whether they are in the matching or the non-matching region. However, for *x*’, in the non-matching region, wildcards in the generated stem *t* are assumed to be matches between *t* and *x*’ while in the matching region they are still treated as mismatches between *t* and *x*’. The number of wildcards is dependent on the Hamming distance between *x* and *x*’ and *d*. Let *HD*(*x*,*x*^′^)=*d*_*x*_. Table 1 shows how many wildcards should be placed in different cases.

**Table 1:** Numbers of wildcards

	**Non-matching region**	**Matching region**
*d*_*x*_≤*d*	0≤*i*≤*d*_*x*_	*d*−max(*i*,*d*_*x*_−*i*)
*d*_*x*_>*d*	*d*_*x*_−*d*≤*i*≤*d*	*d*−max(*i*,*d*_*x*_−*i*)

Assume that *i* wildcards are placed in the non-matching region of *x* to form *t*, resulting in *i* mismatches between *t* and *x* and (*d*_*x*_−*i*)mismatches between *t* and *x*’. We consider the following two cases: 

1. *d*_*x*_≤*d*: The number of wildcards *i* can vary from 0 to the size of the non-matching region. To make the total number of mismatches against *x* smaller than *d*, at most *d*−*i* wildcards can be placed in the matching region of *x*. Similarly, to make the total number of mismatches against *x*’ smaller than *d*, at most *d*−(*d*_*x*_−*i*)wildcards can be placed in the matching region of *x*’.

2. *d*_*x*_>*d*: At least *d*_*x*_−*d* wildcards have to be placed in the non-matching region to eliminate the mismatches. Similar to case 1, in the matching region, at most *d*− max(*i*,*d*_*x*_−*i*)wildcards can be placed.

##### Algorithm 1 **MSS1**

In the matching region, *d*− max(*i*,*d*_*x*_−*i*)is an upper bound on the number of wildcards. However, it is not necessary to enumerate all the cases from 0 to *d*− max(*i*,*d*_*x*_−*i*). Similarly, it is not necessary to repeat stems generation for all pairs in *I*. For any *x* let $x_{1}^{i},x_{2}^{i},\ldots ,x_{j}^{i}\in I$ be *x*’s 2*d*-neighbors in sequence *s*_*i*_ (i.e., $I_{i}=\{x_{1}^{i},x_{2}^{i},\ldots ,x_{j}^{i}\}$)and let *O*_*i*_ be the set of motif instances in *s*_*i*_. Then, clearly, *O*_*i*_⊂*I*_*i*_. The motifs form a subset of stems that can be obtained between *x* and each of *O*_*i*_. The motif stems we generate are stems that can be obtained from *l*-mer pairs of $\left((x,x_{1}^{i}),(x,x_{2}^{i}),\ldots ,(x,x_{j}^{i})\right)$. To minimize the number of stems generated from *I*, we have to use the sequence with the smallest *j*.

##### Observation 3

For any *l*-mer *x*, let its 2*d*-neighbors in sequence *s*_*i*_ be $I_{i}=x_{1}^{i},x_{2}^{i},\ldots ,x_{j}^{i}$ (for 1≤*i*≤*n*). Then, the (*l,d*)-motifs are included in the stems set, which is generated from the pairs $\left(x,x_{1}^{i}),(x,x_{2}^{i}),\ldots ,(x,x_{j}^{i}\right)$.

The detailed MSS1 algorithm is given in Algorithm 1 and Algorithm 2.

In lines 2-18 we find the sequence in which *x* has the minimum number of 2*d*-neighbors. Also, if one sequence has no 2*d*-neighbor, the current *l*-mer *x* is skipped (line 12). The stems are generated by placing wildcards in each pair of *x*×*I*_*min*_, as shown in Algorithm 2.

Hamming distance is called *m*^2^*n* times. Therefore, excluding wildcards placement, Algorithm 1 takes *O*(*m*^2^*nl*)time.

Wildcards placement procedure is called (*m*−*l*+1)times and each time |*I*_*min*_|=*m*−*l*+1 in the worst case. Therefore, in this case, wildcards placement (line 4–16)in Algorithm 2 is run *O*(*m*^2^)times. The number of wildcards is no more than *d*. So line 4–16 takes Old time in the worst case. As a result, wildcards placement in MSS1 takes O(m2ld) time. In the best case, wildcards placement procedure is only called once when all other *l*-mers in *s*_1_ have no 2*d*-neigbhors, and *I*_*min*_=1. The best case for line 4–16 is when *d*_*x*_=2*d* and it takes O2dd time (see DISCUSSION for more analysis).

In summary, MSS1 takes *O*(*m*^2^*nl*+|*stems*|)time, where |*stems*| is the total number of stems generated.

##### Algorithm 2 **PlaceWildcards**

#### MSS2 - A speedup Algorithm

**Figure 1:**
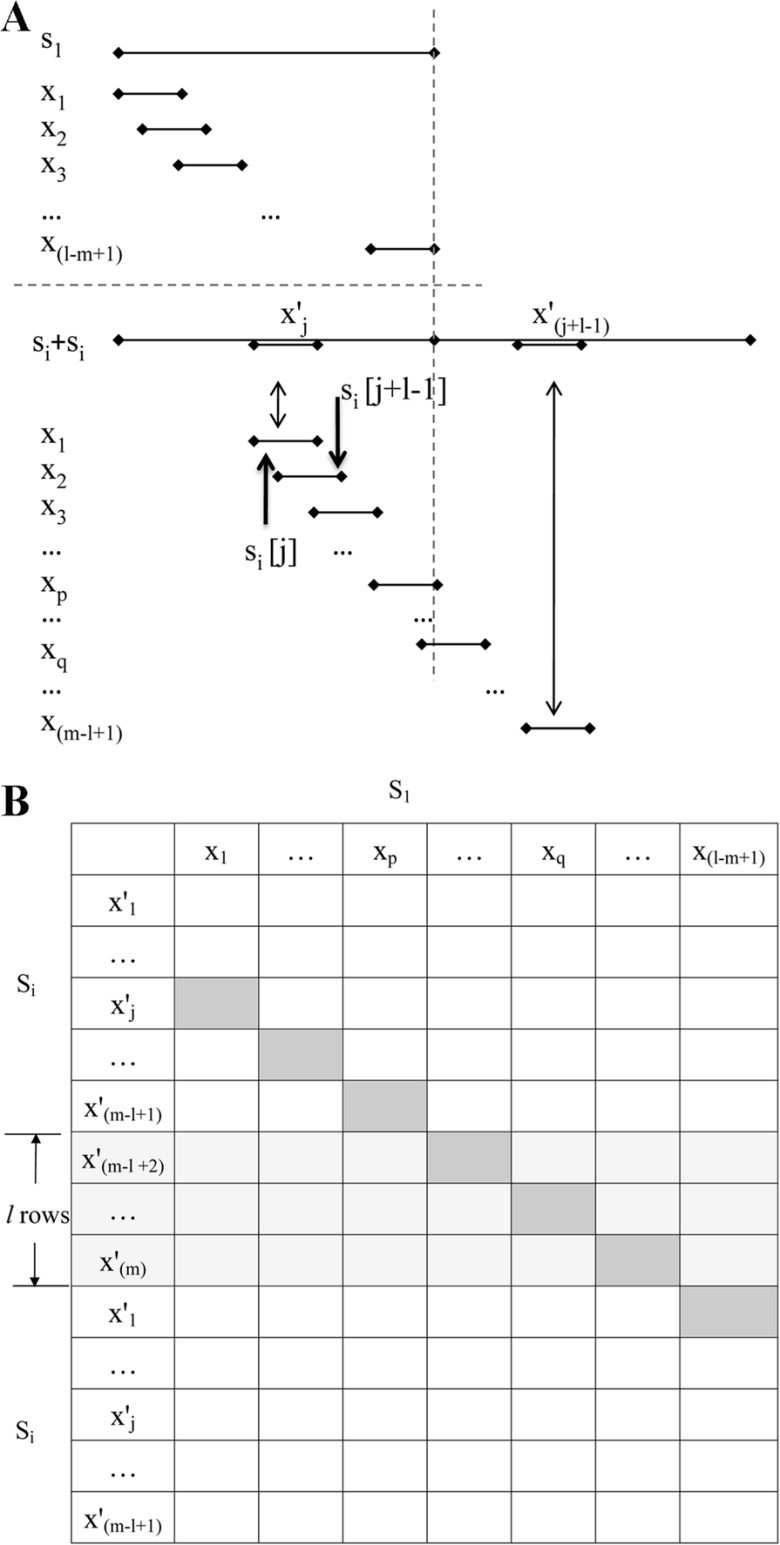
**Illustration of speeding up the 2*d*-neighbors computation. A**: *l*-mer alignments. **B**: computation order in the matrix.

The computation of the 2*d*-neighbors of *x* from *s*_1_ in a certain sequence *s*_*i*_ can be thought of as the calculation of a distance matrix between all (*m*−*l*+1)*l*-mers in *s*_1_ against those in *s*_*i*_ as shown in Figure 1B. A straight forward algorithm takes *O*(*m*^2^*l*)time. When *i* ranges from 2 to *n*, the total time will be *O*(*m*^2^*nl*). In this section we show how to reduce this total time from *O*(*m*^2^*nl*)to *O*(*m*^2^*n*).

Assume that we have computed the Hamming distance between *x*_1_ in *s*_1_ and $x_{j}^{\prime }$ in *s*_*i*_. Let this be $\text{HD}(x_{1},x_{j}^{\prime })=d_{1}$. Then, $\text{HD}(x_{2},x_{j+1}^{\prime })$ can be obtained by comparing: 1)the first characters of *x*_1_ and *x*_*j*_; and 2)the last characters of *x*_2_ and *x*_*j*+1_. Observe that the (*l*−1)-length prefix of *x*_2_ is the (*l*−1)-length suffix of *x*_1_, and $x_{j}^{\prime }$ and $x_{j+1}^{\prime }$ also share the same (*l*−1)-mer.

If the first characters of *x*_1_ and *x*_*j*_ match, then the *d*_1_ mismatches happen in the remaining (*l*−1)-long suffixes of *x*_1_ and *x*_*j*_. In this case, *HD*(*x*_2_,*x*_*j*+1_)=*d*_1_ if the last characters of *x*_2_ and *x*_*j*+1_ match; otherwise *HD*(*x*_2_,*x*_*j*+1_)=*d*_1_+1. Similarly, if the first characters of *x*_1_ and *x*_*j*_ do not match, then there are (*d*_1_−1)mismatches in the remaining (*l*−1)-long suffixes of *x*_1_ and *x*_*j*_. In this case, *HD*(*x*_2_,*x*_*j*+1_)=*d*_1_−1 if the last characters of *x*_2_ and *x*_*j*+1_ match; otherwise *HD*(*x*_2_,*x*_*j*+1_)=*d*_1_. This observation is also mentioned in [4].

##### Observation 4

Given $\text{HD}(x_{1},x_{j}^{\prime })=d_{1}$ where *x*_1_ and $x_{j}^{\prime }$ are two *l*-mers in *s*_1_ and *s*_*i*_, the Hamming distance between the next two *l*-mers of *s*_1_ and *s*_*i*_, $\text{HD}(x_{2},x_{j+1}^{\prime }),$ can be calculated in *O*(1)time as in (1):

(1) $$\text{HD}(x_{2},x_{j+1}^{\prime })=\left\{\begin{array}{ll} d_{1}-1 & \text{if}x_{1}[1]\neq x_{j}^{\prime }[1],x_{2}[l]=x_{j+1}^{\prime }\\ d_{1} & \text{if}x_{1}[1]\neq x_{j}^{\prime }[1],x_{2}[l]\neq x_{j+1}^{\prime }\\ d_{1} & \text{if}x_{1}[1]=x_{j}^{\prime }[1],x_{2}[l]=x_{j+1}^{\prime }\\ d_{1}+1 & \text{if}x_{1}[1]=x_{j}^{\prime }[1],x_{2}[l]\neq x_{j+1}^{\prime } \end{array}\right)$$

However, $\text{HD}(x_{p},x_{q}^{\prime })$ where *p*>*q* is left out when OBSERVATION 4 is used repeatedly and reaches the end of *s*_*i*_. We simply append a copy of *s*_*i*_ to *s*_*i*_ to cover all the pairwise alignments (Figure 1A). Then, by calculating the Hamming distance only once and applying OBSERVATION 4 repeatedly, each diagonal in the matrix of Figure 1B can be computed in *O*(*l*+*m*)time.

We do the above for *m* diagonals from cell $\left(x_{1},x_{1}^{\prime }\right)$ to $\left(x_{1},x_{m}^{\prime }\right)$ in Figure 1B. Then the first and last (*m*−*l*+1)rows are used to form a complete (*m*−*l*+1)×(*m*−*l*+1)matrix. The *l* rows in the middle were eliminated since they are the extra rows caused by appending a copy of *s*_*i*_. Therefore, the computation of the 2*d*-neighbors of *x* from *s*_1_ in any sequence *s*_*i*_ can be computed in *O*(*m*∗(*m*+*l*))=*O*(*m*^2^)time. The computation for all the sequences from *s*_2_ to *s*_*n*_ takes a total of *O*(*m*^2^*n*)time.

The pseudocode is given in Algorithm 3. In lines 6–10, the Hamming distance is calculated for the alignment of *s*_1_ with the *j*_*th*_ position of *s*_*i*_. Each of the remaining Hamming distances in this alignment is obtained in constant time by OBSERVATION 4 (line 12–26). Instead of appending a copy of *s*_*i*_, the *mod* operation is used. **MSS2**

*N*_2*d*_[*k*][*i*] keeps the 2*d*-neighbors of the *k*_*th*_*l*-mer in *s*_1_ in the *i*_*th*_ sequence *s*_*i*_. To build the matrix of 2*d*-neighbors of all the *l*-mers of *s*_1_ (*N*_2*d*_[*k*][*i*]), it takes *O*(*m*^2^*n*)time (lines 3–28). Lines 29–41 search the 2*d*-neighbors of each *l*-mer of *s*_1_. If some sequence *s*_*j*_ has no 2*d*-neighbors, the current *i*_*th*_*l*-mer of *s*_1_ is skipped (lines 32–34). Otherwise, the 2*d*-neighbors in the sequence with the smallest size, *I*_*min*_, are used and the wildcards are placed.

MSS2 takes *O*(*m*^2^*n*+|*stems*|)time.

Optionally, a post-process phase is used following both MSS1 and MSS2 algorithms to refine the output stems. In the post-process phase, a stem is retained only if this stem has at least one neighbor in each sequence at a distance of ≤*d*. This phase takes a total of *O*(*mnl*∗|*stem*|)time.

##### An estimation on the number of stems

We can compute the expected number of stems generated by our algorithms as follows. Let *q* be any *l*-mer in *s*_1_. What can we say about |*I*_*min*_| corresponding to *q*? Consider any sequence *s* other than *s*_1_. Let *Q* be any *l*-mer of *s*. The probability *p* that HD (*q*,*Q*)≤2*d* is ∑i=02dliσ−1σi1σl−i where *σ*=|*Σ*|. This implies that the expected number of such *Q*’s is *mp*. When *Σ* increases, *p* decreases drastically, as examples are shown in Table 2 for *σ*=4 and *σ*=20. In all the previous works (see e.g., [6]), analyses have been done assuming that all the *l*-mers in any sequence are independent. If we assume this, then we can apply Chernoff bounds and show that the number of such *Q*’s is *O*(*mp*)with high probability. This in turn will imply that |*I*_*min*_|=*O*(*mp*)with high probability. *N*_*stems*_, the number of stems generated between any two *l*-mers with the hamming distance *d*_*HM*_, is given in (2), which is crudely bounded by *O*(2^*l*^*l*^*d*^). As a result, it follows that the expected number of stems generated by our algorithms is *O*(*m*^2^*p*2^*l*^*l*^*d*^).

(2) Nstems=∑i=0dHMlild−max{i,dx−i}0≤dHM≤d∑i=dHM−ddlild−max{i,dx−i}d≤dHM≤2d

**Table 2:** **Example values of *p * given | *Σ *|=4 and | *Σ *|=20**

**(*l,d)***	**| *Σ *|=4**	**| *Σ *|=20**
(7,1)	1.29∗10^−2^	6.03∗10^−6^
(9,2)	4.89∗10^−2^	3.32∗10^−5^
(11,3)	1.15∗10^−1^	1.11∗10^−4^
(13,4)	6.38∗10^−2^	8.88∗10^−5^
(15,5)	4.27∗10^−4^	8.22∗10^−7^
(17,6)	5.82∗10^−10^	2.76∗10^−20^
(19,7)	3.64∗10^−12^	1.91∗10^−25^
(21,8)	1.43∗10^−3^	1.21∗10^−5^

###### Algorithm 3 **MSS2**

##### Challenging instances

Consider a PMS instance with *n* sequences of length *m* each. For a given value *l*, let *d* be the smallest integer such that the expected number of (*l,d*)motifs that occur by random chance is ≥1. We refer to (*l,d*)as a challenging instance. We can compute challenging instances as follows. Let the alphabet under concern be *Σ* with |*Σ*|=*σ*. The probability that two random characters in this alphabet match is 1/|*σ*|. Then assuming an IID background, the probability that the Hamming distance between two *l*-mers is no more than *d* is p=∑i=0dliσ−1σi1σl−i. For each sequence, the probability that a random *l*-mer has at least one *d*-neighbor (i.e., an *l*-mer with a Hamming distance of no more than *d*)in this sequence is *P*=1−(1−*p*)^*m*−*l*+1^. This means that the expected number of randomly occurring (*l,d*)motifs in the *n* sequences is *σ*^*l*^*P*^*n*^. From this we can calculate the challenging instances. For *σ*=4, the challenging instances are (7,1),(9,2), etc. When *σ*=20, the challenging instances are (7,4),(9,5), etc. Because of Observation 1, in our tests of challenging instances of protein sequences, we have used the cases of (7,3),(9,4), and (11,5).

## Results

We have evaluated our algorithms on the standard benchmark where *n*=20, *m*=600. Let |*Σ*|=20 (for proteins). We have used (*l,d*)values starting from (7,1)going up to (21,8).

The testing data was generated as follows: 1)20 sequences of length 600 each were generated such that each character in each sequence is equally likely to be one of the characters from the alphabet; 2)a motif of length *l* was generated randomly; 3)a random number of mismatch positions which is smaller than or equal to *d* was selected and the characters in these positions were replaced by other amino acids randomly to form a motif instance; 4)step 3)was done 20 times to generate 20 such instances and these were planted in the 20 sequences (at random places with one instance per sequence).

We have implemented and compared our algorithms with RISOTTO [14] and the stemming algorithm of [21]. Please note that we have implemented the algorithm of [21] since we have no access to a running version of the corresponding program. Both the running time and the number of stems generated were used as performance measures. The machine used had an Intel Core i7-2760QM 2.40GHZ processor with a memory size of 4GB, as shown in Table 3 and Table 4. In these tables "-" indicates that the algorithm took too long to finish. These tables show that MSS1 and MSS2 run faster than RISOTTO [14] and stemming [21]. Since the set of stems is a superset of the true motifs, the stems set contains true motifs and false motifs (or false positive predictions). A smaller number of stems indicates less false predictions. The proposed algorithms generate a much smaller subset of the stems generated by the stemming algorithm [21]. Since instances such as (7,1),(9,2),(11,3),*etc*. are commonly used in DNA sequences, we have also tested the algorithms on more challenging cases such as (7,3),(9,4), and (11,5)as shown in Table 5. In addition to the case of *σ*=20, we have also tried different alphabet sizes: 40, 60, 80, and 100. Table 6 displays the running time for various alphabet sizes. The fact that the rune times are nearly the same for different alphabet sizes indicates that the running time of all the algorithms are independent of the alphabet size. The post-processing phase takes longer time as the alphabet size increases since the number of stems increases.

**Table 3:** Time comparison of MSS, RISOTTO, and stemming algorithms

**(*l,d *)**	**MSS1(s)**	**MSS2(s)**	**Post-process(s)**	**RISOTTO(s)**	**Stemming(s)**
(7,1)	23.2	3.7	0.03	4.7	48.0
(9,2)	24.64	3.7	0.3	242.3	359.9
(11,3)	27.5	3.7	2.0	7166.1	4674.2
(13,4)	34.5	3.9	50.4	-	-
(15,5)	38.8	4.7	74.5	-	-
(17,6)	60.2	15.3	1459.0	-	-
(19,7)	200.8	117.3	18364.1	-	-
(21,8)	719.6	563.2	69340.8	-	-

**Table 4:** Number of stems generated by MSS and stemming algorithms

**(*l,d *)**	**MSS1/MSS2**	**Post-process**	**Stemming**
(7,1)	2	1	928
(9,2)	22	2	17894
(11,3)	130	44	265587
(13,4)	2250	1452	-
(15,5)	5222	1032	-
(17,6)	60168	23829	-
(19,7)	521658	422019	-
(21,8)	2255690	1297576	-

**Table 5:** Comparison of MSS, ROSOTTO, and stemming algorithms on challenging instances

**(*l,d *)**	**MSS1(s)**	**MSS2(s)**	**ROSITTO**	**Stemming**
(7,3)	225.9	615.7	7068.6	>4hours
(9,4)	1051.0	1477.4	>4hours	>4hours
(11,5)	5129.4	5503.0	>4hours	>4hours

**Table 6:** Statistics on different alphabet sizes

**| *Σ *|**	**MSS1(s)/ | *s t e m s *|**	**MSS2(s)/ | *s t e m s *|**	**Post-process(s)/ | *s t e m s *|**	**Stemming(s)/ | *s t e m s *|**
40	25.1/190	3.6/190	2.4/45	2125.5/16669665
60	26.2/400	3.6/400	6.9/169	3023.4/18465345
80	23.6/50	3.6/50	0.4/4	3493.0/11380993
100	27.1/260	3.6/260	5.6/216	4464.9/17733385

Due to better performance, we have used MSS2 in real biological protein data. In Minimotif Miner 3.0 database [1], we randomly sampled 14 protein motifs. Each of these motifs has multiple source proteins. Comparison of MSS, RISOTTO, and stemming is shown in Table 7.

**Table 7:** Motif search on protein data

**Protein motifs**	***#*Source proteins**	**(*l,d *)**	**MSS2(s)**	**RISOTTO(s)**	**Stemming(s)**
CPTINEPCC	7	(9,2)	2.0	100.0	244.0
CRFYNCHHLHEPGC	10	(14,4)	22.2	>4hours	>4hours
HTHPTQTAFLSSVD	8	(14,4)	10.3	>4hours	>4hours
ILPPVPVPK	14	(9,2)	3.8	105.8	582.0
PEPNGYLHIGH	134	(11,3)	51.1	12827.0	>4hours
PSPTGFIHLGN	36	(11,3)	6.5	4336.6	4561.0
PTVYNYAHIGN	19	(11,3)	3.6	3358.9	4917.0
PYANGSIHLGH	110	(11,3)	52.1	11363.2	>4hours
PYPSGQGLHVGH	18	(12,3)	10.4	>4hours	>4hours
QELFKRISEQFTAMF	9	(15,4)	47.6	>4hours	>4hours
QIKTLNNKFASFIDK	9	(15,4)	20.3	>4hours	>4hours
SGYSSPGSPGTPGSR	9	(15,4)	32.6	>4hours	>4hours
SSSSLEKSYELPDGQ	10	(15,4)	41.3	>4hours	>4hours
VTVYDYCHLGH	8	(11,3)	2.9	3145.8	2235.0

Finally, we have compared the MSS2 algorithm with PMSPrune, a well-known Plant Motif Search (PMS)algorithm on DNA sequences [22]. As is clear from Table 8, MSS2 is not as fast as PMSPrune. On DNA sequences, the number of spurious motifs is very large. Therefore, the Motif Stems Search algorithms, which are efficient for large alphabets are not as efficient for small alphabets.

**Table 8:** MSS2 vs. PMSPrune on DNA data

**(*l,d *)**	**MSS2(s)**	**PMSPrune(s)**
(7,1)	4.1	3.3
(9,2)	10.7	3.4
(11,3)	87.2	8.1

## Discussion and conclusions

The analysis in [21] shows that, assuming IID background, the expected number of the (*l*,2*d*)-motifs depends highly on the alphabet size |*Σ*|. Therefore, when |*Σ*| is large, the expected number of 2*d*-neighbors in the *nm*-length sequences is very small in comparison with the total number of *l*-mers (*n*(*m*−*l*+1)).

The proposed algorithms consider an even smaller size of candidates by introducing *I*_*min*_. In particular, for any given *l*-mer *x*, we focus on the sequence that has the smallest number of 2*d*-neighbors for *x*. The expected size of *I*_*min*_ is $\frac{1}{n}$ times the total number of 2*d*-neighbors of *x* in all the sequences. Please note that we do not miss any of the valid motifs.

On the other hand, when generating the stems, as shown in Table 1, once *i* wildcards in the non-matching region are placed, it is known that the upper bound of wildcards in the matching region is *d*− max(*i*,*d*_*x*_−*i*). However, it is not necessary to enumerate all the cases from 0 to *d*− max(*i*,*d*_*x*_−*i*)in the matching region. As long as the case of (*d*− max(*i*,*d*_*x*_−*i*))wildcards cannot be eliminated, 0 to (*d*− max(*i*,*d*_*x*_−*i*)−1)wildcards are contained in the (*d*− max(*i*,*d*_*x*_−*i*))wildcards placement. Therefore, the proposed algorithms do not enumerate 0 to (*d*− max(*i*,*d*_*x*_−*i*)−1)wildcards placements in the output.

In the computation of the 2*d*-neighbors, MSS1 takes *O*(*m*^2^*nl*)time and *O*(*m*)space. MSS2 takes *O*(*m*^2^*n*)time and *O*(*m*^2^)space. The stemming algorithm of [21] uses sorting to compute the set *I*.

The proposed algorithms MSS1 and MSS2 provide an efficient way to solve the Motif Stems Search problem in terms of both time and space. Also, the stems generated by MSS1 and MSS2 form a much smaller subset, with less false predictions, of the stems generated by the algorithm of [21].

## Competing interests

The authors declare that they have no competing interests.

## Authors’ contributions

TM contributed to the implementation of the algorithms, manuscript preparation, algorithms development, and performance analysis. SR contributed to algorithms development, analysis of the results, performance analysis, and manuscript preparation. Both authors read and approved the final manuscript.
